# Biomarkers and the outcomes of ischemic stroke

**DOI:** 10.3389/fnmol.2023.1171101

**Published:** 2023-06-05

**Authors:** Ying Huang, Zhenzhen Wang, Zhi-Xin Huang, Zhenguo Liu

**Affiliations:** ^1^Department of Neurology, Guangdong Second Provincial General Hospital, Guangzhou, Guangdong, China; ^2^Center for Precision Medicine and Division of Cardiovascular Medicine, Department of Medicine, University of Missouri School of Medicine, Columbia, MO, United States

**Keywords:** biomarker, ischemic stroke, prediction, stroke, prognosis

## Abstract

Biomarkers are measurable substances that could be used as objective indicators for disease diagnosis, responses to treatments, and outcomes predictions. In this review, we summarized the data on a number of important biomarkers including glutamate, S100B, glial fibrillary acidic protein, receptor for advanced glycation end-products, intercellular adhesion molecule-1, von willebrand factor, matrix metalloproteinase-9, interleukin-6, tumor necrosis factor-a, activated protein C, copeptin, neuron-specific enolase, tau protein, gamma aminobutyric acid, blood glucose, endothelial progenitor cells, and circulating CD34-positive cells that could be potentially used to indicate the disease burden and/or predict clinical outcome of ischemic stroke. We examined the relationship between specific biomarkers and disease burden and outcomes and discussed the potential mechanisms underlying the relationship. The clinical significance and implications of these biomarkers were also discussed.

## 1. Introduction

Stroke is the second leading cause of death and long-term disability globally and 87% of the stroke patients are classified as ischemic stroke (IS; Saini et al., 2021). The Global Burden of Diseases, Injuries, and Risk Factors Study (GBD) in 2016 showed that IS patients had to bear substantial economic and social burdens including the significant costs of treatment and post-stroke care, and lost productivities (Johnson et al., 2019). Therefore, there is an urgent need to make early diagnosis of IS, identify the etiology, control the risk factors, and improve the outcomes. Currently, the main factors used for risk stratification and prognosis evaluation in patients with IS include advanced age (Chen et al., 2010), gender (Phan et al., 2017), overweight, hypertension, hyperlipidemia, diabetes mellitus, smoking, atrial fibrillation, stroke, and coronary heart disease (Khan et al., 2007), the Essen Stroke Risk Score (ESRS), and the Stroke Prognostic Instrument-II (SPI-II; Weimar et al., 2010; Meng et al., 2011). However, their predictive values are considered modest.

Biomarkers are measurable substances that could be objectively used to aid the disease diagnosis, evaluate the responses to treatments, and predict the outcomes. In this study, we intended to review a few important blood-based biomarkers that are more economical and feasible. Based on their relationship to stroke, these biomarkers can be associated with the disease burden and outcomes of stroke through one or more mechanisms including astrocyte activation, excitotoxicity, endothelial dysfunction, neuroplasticity or neurotrophicity, inflammation, fibrinolysis, metabolic abnormality, and oxidative stress as discussed below and summarized in Table 1.

**Table 1:** Biomarkers associated with the disease burden and outcomes for patients with CVDs.

Mechanism	Biomarker	Description	References	Disease type
Excitotoxicity	Glutamate	The most common excitatory neurotransmitter in CNS; released in large amount from neurons and astrocytes; and acts as an important mediator of neuronal degeneration due to intracellular calcium overload during IS	Zhivotovsky and Orrenius (2011); Chao and Li (2014)	
	GOT	A transaminase enzyme can metabolize glutamate and then reduced edema volume, infarct size, and sensorimotor deficits; high level of blood GOT means favorable outcomes in ischemic stroke patients	Campos et al. (2011a,b,c); Divito and Underhill (2014); Malik and Willnow (2019)	
Astrocyte Activation	S100B	A calcium binding protein from disrupted glial cells and leaked through a damaged blood brain barrier	Herrmann et al. (2000); Ahmad et al. (2012); Michetti et al. (2019)	
	RAGE	A kind of cell membrane bound molecules inducing the productions of pro-inflammatory cytokines in microglia and oxidative stress in neurons after IS	Gasparotto et al. (2019); Dong et al. (2022)	
	GFAP	Intermediate filament protein of astroglial skeleton; leaked from brain tissue in response to brain structural damage	Brenner (2014); van Bodegraven et al. (2019, 2021)	
Endothelial Dysfunction	ICAM-1	Cell surface glycoprotein and an adhesion receptor; regulated by other proinflammatory cytokines associated with early deterioration of ischemic stroke	van de Stolpe and van der Saag (1996); Wang et al. (2006); Bui et al. (2020)	Small vessel disease
	vWF	Released from endothelial cells; plays an important role in adhesion and aggregation of platelets as well as in thrombus formation; increased in IS patients	Menih et al. (2017)	
	CD-34 cells and EPCs	Maintain the homeostasis of vasculature including microcirculation; decreased in the patients with high CVSD burden	Wardlaw (2010)	
Inflammation	MMP-9	Matrix metalloproteinase-9; causing endothelial damage and plaque matrix disruption; released from activated inflammatory cells	Abdelnaseer et al. (2017); Shih et al. (2021); Gross et al. (2022)	Large artery atherosclerosis
	TNF-a	An inflammatory cytokine that induces endothelial necroptosis and subsequent blood brain barrier disruption	Chen A.Q. et al. (2019)	Cardioembolic
	IL-6	An inflammatory cytokine; messenger molecule between leukocytes, vascular endothelium, and parenchymal cells	Kim et al. (1996); Huang et al. (2006); Tuttolomondo et al. (2008)	Cardioembolic
	APC	Endogenous circulating serine proteases with anti-apoptotic, antithrombotic, anti-inflammatory, and neuroprotective qualities	Wang Y. et al. (2013); Mosnier (2019); Sinha et al. (2019)	
Neuroendocrine	Copeptin	A peptide of 39 amino acids; a cleavage product of arginine-vasopressin; reflects the degree of activation of hypothalamic–pituitary–adrenal-axis in IS patients	Jalleh and Torpy (2021); Blek et al. (2022)	
Others	NSE	Neuronal glycolytic enzyme; released from damaged neurons	Missler et al. (1997); Oh et al. (2003); Isgrò et al. (2015)	
	Tau protein	A microtubule-associated protein; maintains the integrity of neuronal cellular skeleton; detectable within 6 h after ischemic symptom onset and peaks after 3–5 days	Bitsch et al. (2002); Pluta et al. (2018); Chen and Jiang (2019)	
	GABA	The most common inhibitory neurotransmitter in CNS; counteracts neuronal excitotoxicity by hyperpolarizing neuron membrane potential and inhibiting glutamatergic transmission	Lyden and Lonzo (1994); Bhattacharya et al. (2014); Liu et al. (2018)	
	Blood glucose	Hyperglycemia is a risk factor of IS; high admission glucose level is associated with END in female patients with IS	Gordon et al. (2018); Wang et al. (2018)	
	AVP	A powerful endogenous hormone that regulates plasma osmolality and volume; a significant rise in blood AVP level is associated with an increase in the size of infarct core area and post-stroke edema	Vakili et al. (2005); Zhao et al. (2015)	

GOT, glutamate oxaloacetate transaminase; CNS, central nervous system; IS, ischemic stroke; RAGE, receptor for advanced glycation end-products; GFAP, glial fibrillary acidic protein; ICAM-1, intercellular adhesion molecule-1; vWF, von Willebrand factor; EPCs, endothelial progenitor cells; CSVD, cerebral small vessel disease; MMP-9, matrix metalloproteinase-9; TNF-a, tumor necrosis factor-a; IL-6, interleukin-6; APC, Activated protein C; NSE, neuron-specific enolase; GABA, gamma aminobutyric acid; and AVP, arginine-vasopressin.

## 2. Pathophysiology of ischemic stroke

Prior to the discussion of specific biomarkers for stroke, it is important to understand the stroke pathophysiology. The “ischemic core” of the ischemic brain tissues is completely cut off from blood supply when vascular occlusion occurs. In the ischemic core, excessive depletion of ATP and failure of aerobic glycolysis often lead to irreversible tissue damage. The region surrounding the core, called the “ischemic penumbra,” is characterized by a significant decrease in cerebral blood flow to the level below functional thresholds. Thus, a metabolic switch to anaerobic metabolism enables neurons and other brain cells in the ischemic penumbra to survive longer despite their functional impairment (Fifield and Vanderluit, 2020). However, cells in the ischemic penumbra could die if cerebral blood flow is not restored in time.

Many time-dependent events occur in the brain during an ischemic stroke, including changes in ion channel function, metabolisms, and cellular activities (Maestrini et al., 2016). Neuronal excitotoxicity and calcium overload occur within minutes of stroke onset as the first early effects of cerebral ischemia. ATP depletion induces the dysfunction of ion channels, leading to the release of glutamate into extracellular compartment (Szydlowska and Tymianski, 2010). Over-activated glutamate receptors increase intracellular calcium, sodium, and water influx, and activate catabolic processes in cells (Belov Kirdajova et al., 2020). Following calcium overload, calcium-dependent enzymes are activated, which results in the formation of superoxide, nitric oxide, and arachidonic acid metabolites (Ludhiadch et al., 2022). The next phase of response or delayed response to ischemia is characterized by inflammation in the ischemic brain tissue within hours of stroke. Cerebral vasculature is the first to be affected by the ischemic insult and develops an inflammatory response to the insult. Thus, the perivascular microglia macrophages release a variety of cytokines, including tumor necrosis factor (TNF)-a and interleukin (IL)-1b, and initiate the inflammatory response with releases of IL-8 and IL-6, resulting in sustained inflammation with increased levels of fibrinogen and C-reactive protein (CRP), and upregulation of cell adhesion molecules including immunoglobulin superfamily [such as intercellular adhesion molecule type 1 (ICAM-1) and vascular adhesion molecule type 1 (VCAM-1)], selectins (such as P- and E-selectin), and integrins (Anrather and Iadecola, 2016; Khoshnam et al., 2017; Sakai and Shichita, 2019). Both microglia and astrocytes play a dual role in IS, with two polarization states: the pro-inflammatory phenotype (M1 and A1) and the anti-inflammatory phenotype (M2 and A2; Qin et al., 2019). Microglia are activated immediately to become M1 phenotype in response to IS, simultaneously promoting astrocyte activation to A1 phenotype. As the disease conditions progress, the microenvironment changes and the reactive astrocytes transform to M2 phenotype that crosstalk with A2 (Liu et al., 2020).

**Figure 1:**
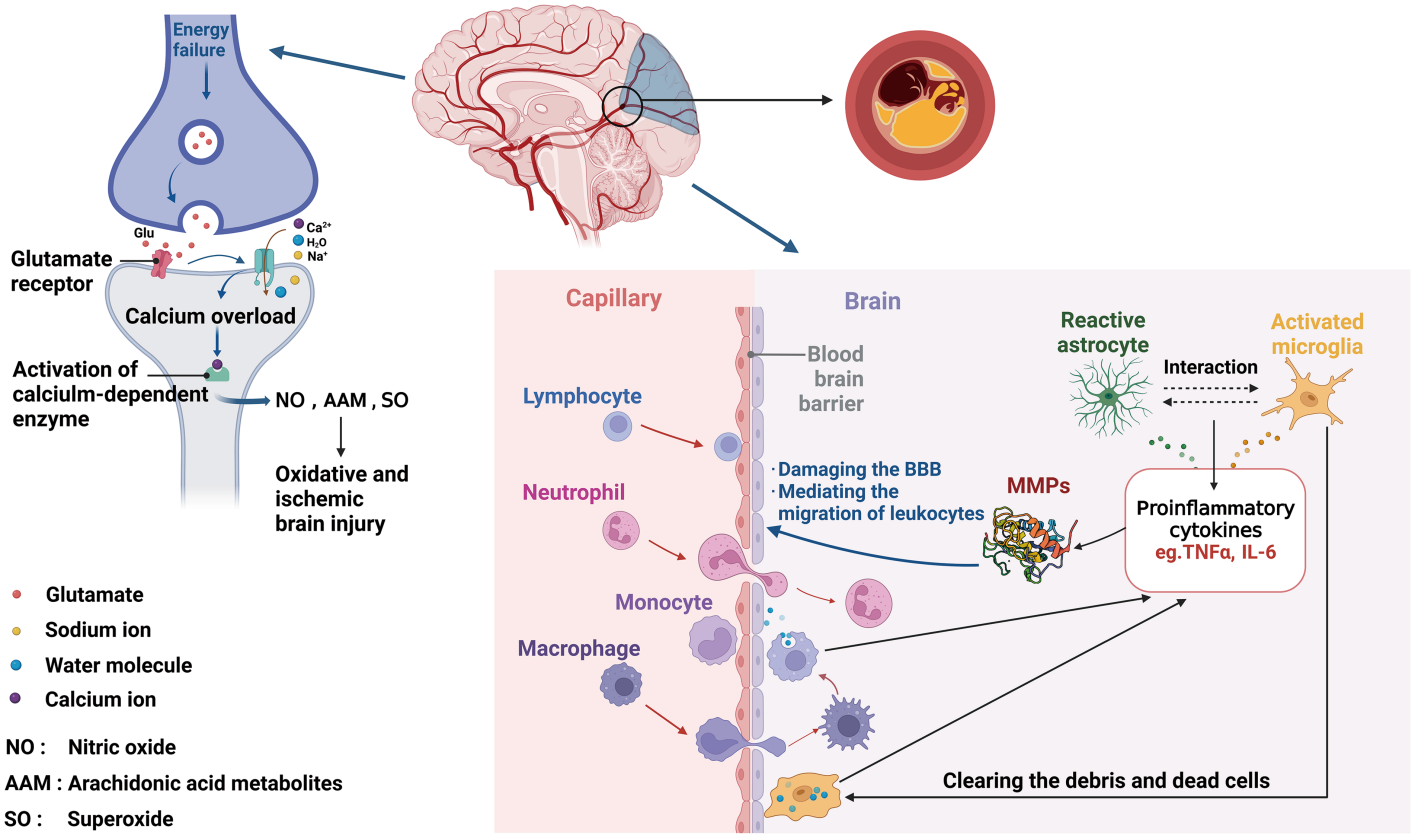
Involvement of biomarkers in ischemic stroke pathophysiology. After ischemic events, neurons suffer from energy failure, inducing the dysfunction of ion channels, leading to the release of glutamate, then increasing intracellular calcium, sodium, and water influx. Following calcium overload, calcium-dependent enzymes are activated, which results in the formation of NO, AAM, and SO, ultimately leading to oxidative and ischemic brain injury. Within the neurovascular unit, microglia get the activated response immediately after IS, with releasing proinflammatory cytokines (TNFɑ, IL-6, and MMP), simultaneously promoting astrocyte activation. Reversely, reactive astrocytes activate microglia, likewise secreting various factors, which damage the BBB, then inducing the migration of peripheral immune cells, leading to secondary BBB damage.

Immediately after stroke, neutrophils are recruited to the brain, followed by monocytes and lymphocytes within 48 h (Fifield and Vanderluit, 2020; Kollikowski et al., 2020; Kömürcü et al., 2020). Proinflammatory cytokines, such as IL-6 and TNF-a, induce the production of matrix metalloproteinase (MMP), especially MMP-9. MMPs then disrupt blood–brain barrier (BBB), and promote the migration of leukocytes, which then interact with endothelium and adhere to vascular walls (Kurzepa et al., 2005; Polavarapu et al., 2005). Edema and hemorrhagic transformation (HT) may arise because of BBB disruption (Wang et al., 2020). Macrophages derived from blood and microglia accumulate in the border zone to clear dead cells and debris, generating proinflammatory mediators and cytotoxic molecules (Sawada, 2009). Cell apoptosis and necrosis are caused by calcium overload, which can persist for hours or days after stroke onset (Ludhiadch et al., 2022; Figure 1).

Therefore, a significant number of cell populations and/or bioactive substances are closely associated with various phases of disease process after stroke. Thus, these cell populations and/or bioactive substances could serve as biomarkers for disease burden and/or clinical outcomes of stroke. According to the pathophysiology of ischemic stroke, these potential biomarkers can be classified as markers or indicators relating to excitotoxicity, endothelial dysfunction, astrocyte activation, inflammation, fibrinolysis, neuroplasticity or neurotrophy, metabolic abnormality, and oxidative stress as discussed and summarized in Table 1 below.

## 3. Specific biomarkers for disease burden and outcomes for patients with IS

### 3.1. Biomarkers associated with excitotoxicity

#### 3.1.1. Glutamate

Glutamate is one of the most common excitatory neurotransmitters in the central nervous system (CNS) in a healthy subject. It is well known that, during ischemia, glutamate is released in massive amounts from neurons and astrocytes, and acts as an important mediator of neuronal degeneration due to cellular overload of calcium primarily caused by calcium-permeable NMDA receptor (Chao and Li, 2014; Kaplan-Arabaci et al., 2022). As a result of calcium overload, cells necrotize and phospholipids, proteins, and DNA are broken down (Zhivotovsky and Orrenius, 2011). Disruption of energy metabolism and failure of ion channel functions as well as dysfunction of reuptake mechanisms in stroke largely contribute to glutamate accumulation in the extracellular space, making the brain vulnerable to ischemic insult. Glutamate overload in turn leads to prolonged stimulation of NMDA and AMPA ionotropic receptor subtypes, and dramatically enhances calcium, water, and sodium influx into neurons, thus activating intracellular enzymes and triggering neuronal death (Saha et al., 2021). In addition, cortical spreading depolarization (CSD), a high energy consuming electrical activity with a wave of neuronal and glial depolarization across the brain’s gray matter, is triggered by high glutamate and K^+^ levels extracellularly, resulting in slowly propagating massive depolarization of astrocytes and neurons accompanied by drastic disruptions of ionic gradients.

Studies have revealed that neurologic deterioration of patients with ischemic stroke is positively associated with higher glutamate levels in blood and cerebrospinal fluid (Castellanos et al., 2008). Human plasma glutamate levels correlate with ischemic lesions on CT scans or MRI and neurological outcomes during the first 24 h after stroke (Meng et al., 2015). It has been reported that an increased blood glutamate level is closely related to the development of acute lung injury (ALI), a serious complication of stroke, and an indicator of poor prognosis after stroke (Bai et al., 2017).

#### 3.1.2. Glutamate oxaloacetate transaminase

Glutamate oxaloacetate transaminase (GOT) is a transaminase enzyme that is normally expressed abundantly in the liver and cardiomyocytes (Campos et al., 2011a; Rink et al., 2011). Using oxaloacetate as co-substrate, GOT converts glutamate to 2-ketoglutarate and aspartate. This enzyme can metabolize glutamate in the peripheral blood as well. Under physiological conditions, glutamate is released in synaptic cleft and then quickly removed by the excitatory amino acid transporters (EAATs) in astrocytes and neurons (Divito and Underhill, 2014; Malik and Willnow, 2019). Besides astrocytes and neurons, EAATs have been identified in the endothelial cells of brain vasculature (Hawkins et al., 2006; Hawkins, 2009), indicating a potential important role of vascular endothelial cells in the regulation of glutamate dynamics. In fact, it has been described that glutamate carriers are present in the luminal membrane of endothelial cells and EAATs in the abluminal membrane (Cohen-Kashi-Malina et al., 2012; Hinca et al., 2021). The distributions of glutamate carriers and EAATs in endothelial cells could allow glutamate diffusion from brain tissue to blood following its concentration gradient. Therefore, a decrease in blood glutamate level could reduce the extracellular levels of glutamate in brain (Teichberg et al., 2009). In an animal model of cerebral ischemia, oxaloacetate-mediated GOT activation induced a decrease in blood and cerebral glutamate levels that reduced edema volume, infarct size, and sensorimotor deficits (Campos et al., 2011b). Similarly, clinical studies have revealed a positive association between high levels of blood GOT and favorable outcomes in ischemic stroke patients (Campos et al., 2011c).

### 3.2. Biomarkers associated with astrocyte activation

#### 3.2.1. S100B

The 21-kDa Ca2^+^-binding protein S100B is expressed mainly in astrocytes and Schwann cells of the central nervous system (Michetti et al., 2019). S100B levels in peripheral tissues after ischemic stroke have been significantly elevated in patients and animals (Choi et al., 2020; Onatsu et al., 2020; Kadirhan et al., 2022). Mechanistically, previous studies suggested that S100B could be released from activated and/or disrupted glial cells (including cells within ischemic brain tissue) into cerebrospinal fluid (CSF), and with subsequent leaking into the bloodstream via a ruptured blood–brain barrier (BBB; Herrmann et al., 2000; Ahmad et al., 2012).

Data from six longitudinal studies has demonstrated that, although serum levels of S100B are not raised immediately following acute ischemic stroke, a spike of serum S100B levels occurs 3 days after symptom onset of stroke. The data has also shown that serum S100B levels are closely associated with infarct volume and are positively correlated with the risk of malignant infarction or hemorrhagic transformation after thrombolysis as well as with functional outcome in stroke patients (Dassan et al., 2009). Thus, serum S100B levels may be used as a promising marker to identify patients at increased risk of specific early neurological complications like hemorrhagic transformation after stroke and as an indicator of cerebral damage and functional outcome (Foerch et al., 2007). However, further studies are needed to establish the predictive values of serum S100B levels for disease burden and clinical outcomes in stroke patients.

#### 3.2.2. Receptor for advanced glycation end-products

The receptors for advanced glycation end-products (RAGE) are cell membrane bound molecules, and expressed in glia, neurons, and microglia in human cortex and hippocampus (Gasparotto et al., 2019). In addition, RAGE mediates amyloid beta transport across the blood–brain barrier into the CNS, inducing the productions of pro-inflammatory cytokines in microglia and oxidative stress in neurons (Dong et al., 2022). The soluble isoforms of RAGE (sRAGE) represent the extracellular domain of RAGE without cytosolic and trans-membrane domains. Two isoforms of sRAGE exist: cleaved RAGE (cRAGE) and endogenous secretory RAGE (esRAGE). Recently, there is evidence that cRAGE may be the main form of circulating sRAGE after ischemic stroke (IS), and it may independently predict a poor prognosis. It has been shown that plasma sRAGE level is significantly increased in IS patients shortly after the event (<48 h after onset) and then decreased in 5–7 days (Yang et al., 2018; Aida et al., 2021; Zhang et al., 2022).

#### 3.2.3. Glial fibrillary acidic protein

Glial fibrillary acidic protein (GFAP) is a monomeric intermediate filament protein of astroglial skeleton with a molecular weight of between 40 and 53 kDa and is considered brain specific (van Bodegraven et al., 2019, 2021). GFAP is found in the white and gray matters in CNS and is released rapidly from damaged brain tissues (Brenner, 2014; Griffin et al., 2019). Serum GFAP concentrations have been reported to be increased with the highest values at 48 h after stroke onset and remains elevated for at least 5 days after stroke (Wunderlich et al., 2006). In addition, the kinetics of GFAP release are associated with the development and progression of neurological deficits and infarction, and thus may be potentially used as an additional indicator of the early and functional outcomes (Puspitasari et al., 2019). Since the serum levels of GFAP are normally low, structural damage to brain causes a leakage of GFAP from brain tissue to CSF regardless of BBB functional status. Data have shown that serum GFAP levels are associated with poor outcomes after adjustment for stroke severity and infarct volume (Chen et al., 2020; Pujol-Calderón et al., 2022). However, other mechanisms should be considered for increased levels of serum GFAP, including (1) as GFAP is an intermediate filament of the cytoskeleton, it plays an important role in reactive astrogliosis of the CNS (Eng, 1985; Sims and Yew, 2017); (2) functional changes of astrocytes in response to damages to nerve tissues, metabolic abnormalities, and status of neurodegeneration (Siracusa et al., 2019; Li et al., 2022); and (3) response to proinflammatory cytokines (Giovannoni and Quintana, 2020).

### 3.3. Biomarkers associated with endothelial dysfunction

#### 3.3.1. Intercellular adhesion molecule-1

Intercellular adhesion molecule-1 (ICAM-1, also known as CD54) is a cell surface glycoprotein that is overexpressed in vascular endothelial cells and epithelial cells in response to a variety of inflammatory mediators and activation of the immune system (van de Stolpe and van der Saag, 1996; Bui et al., 2020). Various cytokines produced during the inflammatory process significantly upregulate the expression of ICAM-1, thereby strengthening the adhesion between vascular endothelial cells and activated immune/epithelial cells (Haraldsen et al., 1996; Stanimirovic et al., 1997). ICAM-1 has been shown to be involved in acute inflammation during reperfusion injury, resulting in tissue destruction and brain damage after acute ischemia and subarachnoid hemorrhage. A strong correlation has been reported between prognosis and the soluble adhesion molecules concentrations in the patients with acute spontaneous intracerebral hemorrhage (ICH; Wang et al., 2011). A multivariate analysis has shown that sICAM-1 (soluble intercellular adhesion molecule 1) serum levels on admission are associated with neurological deterioration in stroke patients (Wang et al., 2006).

There is still no explanation for the association between ICAM-1 and neurological deterioration following an ischemic stroke. There are currently three hypotheses: (1) as a vital adhesion molecule, ICAM-1 mediates the neutrophil recruitment and migration effectively, and thus significantly contributing to the edema and inflammatory responses in ischemic brain (Clark et al., 1993; Lindsberg et al., 1996; Simundic et al., 2004). Thus, ICAM-1 level would be expected to be associated with the degree of inflammatory responses and cerebral edema in the lesions. However, a relationship of periphery ICAM-1 levels with the levels of inflammation in CNS in ischemic stroke remains to be established. (2) In early stages of ischemic stroke, ICAM-1 expression is partly regulated by other proinflammatory cytokines (Lee et al., 2000; Frank and Lisanti, 2008; Siniscalchi et al., 2014). Proinflammatory cytokines are directly responsible for increasing ICAM-1 expression in endothelial cells, promoting leukocyte-endothelium interactions, and activating local endothelium to be prothrombotic (Stanimirovic et al., 1997; Zhang, 2008; Maloney and Gao, 2015). And (3) ICAM-1 might be related to post ischemic stroke thrombosis since a recent study has suggested the involvement of ICAM-1 in coagulation (Wang et al., 2006).

#### 3.3.2. Von Willebrand factor

Von Willebrand factor (VWF) is predominantly produced and released from endothelial cells and plays an important role in adhesion and aggregation of platelets as well as in thrombus formation. An increased level of VWF indicates a significant endothelial injury or dysfunction. VWF is a major factor for accumulation and activation of platelets in stenotic arteries, leading to acute thrombotic occlusion (Menih et al., 2017). It has been suggested that high levels of VWF could be associated with increased severity of stroke as well as with poor clinical outcome. Elevated VWF levels immediately after lysis and at 24 h post-therapy have been shown to be an independent prognostic predictor for poor functional outcomes at 90 days for patients with ischemic stroke (Toth et al., 2017). By breaking down VWF, a disintegrin and metalloprotease with thrombospondintype 1 motif, member 13 (ADAMTS13) has been shown to reduce inflammatory reaction and promote ischemic vascular remodeling (Xu et al., 2017). The recombinant ADAMTS13 may potentially become a novel and effective pharmacological agent (Wang L. et al., 2013; Nakano et al., 2015; Chen X. et al., 2019).

#### 3.3.3. CD-34 cells and endothelial progenitor cells

There are three main causes of cerebral small vessel disease (CSVD): lacunar infarction, cerebral microbleeds, or white matter hyperintensities (Wardlaw et al., 2013b; Hilal et al., 2017). Uncertainty exists regarding how CSVD develops and progresses. Brain function declines as a result of abnormalities in cerebral microcirculation related to cerebral microvascular disease (Hilal et al., 2017; Wardlaw et al., 2019). Microcirculation, as well as the vasculature, relies heavily on endothelial cells (Diaz-Otero et al., 2017). Vascular structural and functional integrity is influenced by circulating endothelial progenitor cells (EPCs), which promote endothelial regeneration/repair and contribute to new vascular formation (Tesfamariam, 2016). Moreover, EPCs play a critical role in the integrity of the brain’s blood–brain barrier (BBB) and neurovascular integrity (Rouhl et al., 2008; Huang et al., 2013). Pathogenesis of CSVD is believed to be influenced by BBB dysfunction (Wardlaw, 2010; Wardlaw et al., 2013a). A recent study of 364 patients with CSVD showed that the odds ratio (OR) for subjects with a high CSVD burden was 0.42, and circulating CD34^+^CD133^+^CD309^+^ and CD34^+^CD133^+^ cells were significantly higher for patients with a low CSVD burden (Huang et al., 2021). According to these findings, circulating CD34^+^ cells, CD34^+^CD133^+^ cells, and CD34^+^CD133^+^CD309^+^ cells may serve as biomarkers to track CSVD disease progression.

### 3.4. Biomarkers associated with inflammation

#### 3.4.1. Matrix metalloproteinase-9

The matrix metalloproteinase-9 (MMP-9) belongs to the zinc-dependent MMP family and is essential for extracellular matrix remodeling (Shih et al., 2021; Gross et al., 2022). During cerebral ischemia, MMP-9 expression increases significantly, which is associated with BBB disruption, edema formation, or hemorrhage. As a result of neuronal cell death during an ischemic stroke, inflammation cells infiltrate and activate. The activated inflammatory cells in turn release cytotoxic substances including MMPs, inducing further cell damage and BBB disruption (Abdelnaseer et al., 2017). It has been reported that, although elevated serum levels of MMP-9 in patients with acute ischemic stroke are positively correlated with poor stroke outcomes in the first 24 h after onset of symptoms (Abdelnaseer et al., 2017), the serum MMP-9 level after 1 month of stroke could serve as a positive predictor of stroke recovery beyond the acute stage (Abdelnaseer et al., 2017). Another study also showed that an increase in serum MMP-9 levels during the acute phase of ischemic stroke contributed to an increased mortality and major disability rate (Kots and Weinstein, 2021), suggesting that serum MMP-9 could be a significant predictor of ischemic stroke prognosis.

#### 3.4.2. Tumor necrosis factor-a

Proinflammatory cytokines can activate inflammatory pathways, leading to increased productions (either locally or systematically or both) of nitric oxide, adhesion molecules, TNF-a, and interleukin (IL)-6, thus, contributing to neurotoxicity/neuroprotection (Maestrini et al., 2016). In addition, it is shown that TNF-α from M1 type microglia and TNF receptor 1 (TNFR1) in endothelial cells are critically involved in mediating perivascular M1-like microglia-induced endothelial necroptosis and subsequent BBB disruption (Chen A.Q. et al., 2019). The levels of TNF-a in CSF have been found to be increased in patients at 24 h, 1 week, and 2 weeks after stroke, and correlate with infarct volume and severity of neurological impairment (Shang et al., 2020; Deng et al., 2021). Real-time PCR analysis in rats after t-MCAO has revealed that the mRNA levels for TNF-a, IL-1b, IL-6, E-selectin, and ICAM-1 are increased 3 h after stroke that persist for 24 h in the hemisphere ipsilateral to occlusion (Elango and Devaraj, 2010; Luo et al., 2021).

#### 3.4.3. Interleukin-6

Interleukin-6 (IL-6) is an important inflammatory cytokine that is involved in the pathogenesis of various neurological disorders including strokes by acting as a messenger molecule between vascular endothelium, leukocytes, and parenchymal cells (Kim et al., 1996; Huang et al., 2006; Tuttolomondo et al., 2008). It has been shown that IL-6 is a critical mediator of cerebral ischemia-induced inflammatory responses (Dziedzic et al., 2004; Zeng et al., 2013). In experimental models of brain ischemia, IL-6 can be detected in nerve tissue as early as an hour after the middle cerebral artery (MCA) is occluded (Choi et al., 2010; Maddahi and Edvinsson, 2010). In a cross-sectional descriptive study of 31 acute stroke patients, IL-6 levels correlated significantly with NIHSS and mRS scores in the patients from time of admission to the end of the follow-up period (Mosarrezaii et al., 2020). However, large studies are needed to confirm the finding and to determine if serum IL-6 levels could be used as a predictor of prognosis for patients with ischemic strokes.

#### 3.4.4. Activated protein C

Endogenous circulating serine proteases such as activated protein C (APC) exhibit anti-apoptotic, antithrombotic, anti-inflammatory, and neuroprotective qualities (Mosnier, 2019; Sinha et al., 2019). The protease activated receptor 1 (PAR1) is an important receptor for APC-mediated cell-signaling in many types of CNS and periphery cells. Within the neuro vascular unit, APC activates neuroprotective, vasculoprotective, and anti-inflammatory pathways in brain neurons, microglia, and endothelium, thus, protecting CNS after acute or chronic injuries (Wang Y. et al., 2013), by blocking p53-mediated apoptosis in ischemic human brain endothelial cells (Mendioroz et al., 2009).

### 3.5. Biomarkers associated with neuroendocrine system—Copeptin

A glycopeptide of 39 amino acids, copeptin is a fragment of the prohormone pre-pro-vasopressin, which is a precursor to vasopressin. Inside the posterior pituitary gland, pre-pro-vasopressin is cleaved into copeptin and vasopressin, which are circulating in equimolar amounts (Blek et al., 2022). The serum copeptin level is a marker of vasopressin secretion due to its high stability, and reflects the degree of activation of hypothalamic–pituitary–adrenal-axis in IS patients (Jalleh and Torpy, 2021). A study has demonstrated that a 24-h plasma copeptin levels after stroke onset are related to mortality and unfavorable functional outcome at 1 year (Spagnolello et al., 2019). Recently, a new prognostic score system “the CoRisk score” was developed for patients with acute ischemic stroke with improved predictive power, based on the blood marker copeptin (de Marchis et al., 2019).

### 3.6. Others

#### 3.6.1. Neuron-specific enolase

Neuron-specific enolase (NSE) is an intracellular enzyme in neurons and neuroendocrine cells (Isgrò et al., 2015). It is released into the extracellular medium when neuron death occurs, thus making it possible to assess the extent of structural impairment of the biomembranes in CNS (Missler et al., 1997; Oh et al., 2003), serving as a marker of neuron damages. In addition, upon stimulation, cell surfaces can be damaged by NSE as it acts as a plasminogen receptor and induce tissue damage and extracellular matrix degradation, production of pro-inflammatory chemokines/cytokines, and accumulation of inflammatory cells at inflamed and injured sites (Haque et al., 2016). Recently, Kurakina et al. (2021) reported that, along with the severity of neurological symptoms below the score of 15 (NIHSS), a blood NSE concentration of less than 2 ng/mL in patients with acute ischemic stroke at the acute phase predicts a good functional outcome at 12–14 days after disease onset.

#### 3.6.2. Tau protein

Tau protein, a microtubule-associated protein, is mainly expressed in the brain, and to a lesser extent in the heart, lung, kidney and testis (Pluta et al., 2018). It helps to maintain the integrity of neuronal cellular structure. Tau protein is also involved in the regulation of apoptosis, oxidative stress, autophagy, excitotoxicity, angiogenesis, endothelium and inflammation, and mitochondrial function of the brain tissues (Chen and Jiang, 2019). It was observed that, within 6 h after ischemic symptom onset, measurable amount of tau protein was present in the serum and reached its concentration after 3–5 days, then returned to baseline value 3 months later (Bitsch et al., 2002). Test for Tau protein in the serum of patients with ischemic stroke is now considered a potential prognostic factor for more severe neurological deficits and worse functional outcomes in early and late phase of ischemic stroke (Bielewicz et al., 2011).

#### 3.6.3. Gamma aminobutyric acid

Gamma aminobutyric acid (GABA) is a major inhibitory neurotransmitter in CNS (Liu et al., 2018). At the time when glutamate is released in the cerebral regions due to ischemic insult, GABA might be released simultaneously to counteract the neuronal excitotoxicity by hyperpolarizing neuron membrane potential and blocking glutamatergic transmission (Lyden and Lonzo, 1994; Bhattacharya et al., 2014). It has been demonstrated that GABA levels of <240 nmol/L had a positive predictive value for neurological deterioration in acute lacunar infarctions (Serena et al., 2001). There are currently active investigations on therapeutic drugs targeting GABA and its receptor (agonists), such as muscimol, diazepam, and chlormethiazole, to improve stroke outcomes (Serena et al., 2001; Liu et al., 2018).

#### 3.6.4. Blood glucose

Those with hyperglycemia are more likely to suffer a stroke, regardless of whether they have diabetes or not (Gordon et al., 2018; Wang et al., 2018). Acute ischemic stroke (AIS) patients with early neurological deterioration (END) have a poor prognosis (He et al., 2019). It has been found that male patients with acute ischemic stroke have a significantly higher risk for END than female patients, and blood glucose levels at admission are independently associated with END. Subgroup analysis, however, showed that admission glucose levels were significantly related to increased risk of END only for females. An admission blood glucose level of 107.1 mg/dL had a significant predictive value for END prediction, with a specificity of 53% and a sensitivity of 100% in females (Huang et al., 2020). Researchers found that END development in AIS patients might be sex-dependent, with males at a higher risk, and admission glucose levels might be a significant predictor for END in female patients with AIS.

#### 3.6.5. Arginine-vasopressin

Brain edema after stroke is closely related to arginine-vasopressin (AVP), a powerful endogenous hormone that regulates plasma osmolality and volume (Vakili et al., 2005). A significant rise in blood AVP level is associated with an increase in the size of infarct core area and post-stroke edema (Zhao et al., 2015). Although the value of AVP level as a biomarker for predicting the outcome of stroke has yet to be established, experimental therapy using conivaptan, a mixed vasopressin antagonist, has been tested in stroke patients for brain edema management safely (Hedna et al., 2014).

## 4. Significance and clinical implications

Biomarkers have been playing important roles in disease diagnosis and progression as well as evaluations of treatment response, side/adverse effects, and clinical outcomes for many disease conditions including cardiovascular diseases and cancers. To date, a number of biomarkers of CVDs have been identified, and their relationships with CVDs have been studied to some extent. Some of the biomarkers are specific to neurological system such as NSE, copeptin, and tau protein. However, the use of these markers has not been widely used clinically for predicting the outcomes of stroke very likely due to their limited sensitivity and/or specificity for a single biomarker. Recently, efforts have been made to use a panel of biomarkers to provide more prognostic information on clinical outcomes and function than using a single biomarker (Zhong et al., 2019). A combination of multiple biomarkers (including complement C3, high-sensitive C-reactive protein, hepatocyte growth factor, matrix metalloproteinase-9, and anti-phosphatidylserine antibodies) could substantially improve the risk stratification of adverse outcomes in ischemic stroke patients (Haque et al., 2016).

In addition to conivaptan, a few agents have been under development to target MMP9, including luteolin (Luo et al., 2019), carnosine (Kim et al., 2021), and memantine (Beladi Moghadam et al., 2021), to protect against cerebral ischemic injury.

## 5. Conclusion

There are many biomarkers for disease burden and clinical outcomes of CVDs. However, the value of most identified biomarkers as a predictor of clinical outcomes or in the management of stroke patients to improve patient’s outcomes remains uncertain. Systematic and well-designed large clinical studies are needed to establish the predictive values of each biomarker and/or their combinations on disease burden and clinical outcomes for patients with CVDs.

## 6. Limitations

Many epidemiological studies suggest that genetic susceptibility might be associated with stroke (Meschia et al., 2011; Traylor et al., 2021). In this review, no genetic biomarkers were discussed due to their complexity and needs of further validations.

## Author contributions

Z-XH and ZL contributed to the conception and design of the study. YH wrote the first draft of the manuscript. ZW and Z-XH designed the figure. Multiple revisions and additions to the final manuscript are contributed by YH, ZL, and Z-XH. All authors contributed to the article and approved the submitted version.

## Funding

This work was partially supported by the Science and Technology Program of Guangzhou, China (202201020359).

## Conflict of interest

The authors declare that the research was conducted in the absence of any commercial or financial relationships that could be construed as a potential conflict of interest.

## Publisher’s note

All claims expressed in this article are solely those of the authors and do not necessarily represent those of their affiliated organizations, or those of the publisher, the editors and the reviewers. Any product that may be evaluated in this article, or claim that may be made by its manufacturer, is not guaranteed or endorsed by the publisher.
